# Adaptation through Collaboration: Developing Novel Platforms to Advance the Delivery of Advanced Therapies to Patients

**DOI:** 10.3389/fmed.2017.00056

**Published:** 2017-05-29

**Authors:** Magdalini Papadaki

**Affiliations:** ^1^Association of the British Pharmaceutical Industry, London, United Kingdom

**Keywords:** gene therapy, cell therapy, ATMP, open innovation, precompetitive collaboration, accelerated pathways

## Abstract

For the nascent field of advanced therapies, collaboration will be a game-changer, turning scientific progress that was once unimaginable into transformative medical practice. Despite promise for lifelong management and even cure of disease, skepticism remains about the feasibility of their delivery to patients, fueling investment risks. With the potential for long-term effectiveness in need of frequent reassessment, current approaches to predict real-life drug performance bear little relevance, necessitating novel and iterative schemes to monitoring the benefit–risk profiles throughout the life span of advanced therapies. This work explains that reinventing an adoption route for Advanced Therapy Medicinal Products is as much about the scientific and clinical components, as it is about the organizational structures, requiring an unprecedented level of interactions between stakeholders not traditionally connected; from developers and regulators, to payers, patients, and funders. By reflecting on the successes and lessons learned from the growing space of global precompetitive consortia and public–private partnerships, as well as a number of emerging accelerated development pathways, this work aims to inform the foundations for a future roadmap that can smooth the path to approval, reimbursement, and access, while delivering value to all stakeholders. Echoing the growing demands to bring these transformative products to patients, it provides critical insights to enhance our capacity in three fundamental domains: deploying the operational flexibilities offered by the growing space of collaborations, utilizing emerging flexible and accelerated pathways to tackle challenges in quantifying long-term effectiveness, and building the necessary digital and clinical infrastructure for knowledge development.

Advanced Therapy Medicinal Products (ATMPs), including cell/gene therapies and tissue engineered products (1), offer unprecedented promise for long-term management and even cure of disease, especially in areas of high-unmet medical need, from terminal forms of cancer to vision loss. The socioeconomic and patient benefits of building an ATMP enterprise could be immense, reflected by the recent volume of investment, and mushrooming number of clinical trials in gene therapies for rare diseases and immuno-oncology. However, skepticism remains about the feasibility of their commercialization and delivery to patients, especially as the durability of their effect can only be determined in the long haul (2).

Notwithstanding a number of technical and development challenges, generating sufficient clinical and cost-effectiveness data, achieving reimbursement, and embedding them to existing medical practice remain opaque. In addition, ATMPs are tested by the broader inefficiencies of the current system, which remains expensive and slow in getting affordable new therapies to the right patients at the right time, fueling the need for a paradigm shift.

The common denominator for traditional drugs and ATMPs involves achieving a trade-off between the need for timely access and robust evidence of clinical and economic outcomes. In contrast to the current binary, pre/post-market model of clinical and commercial assessment, removing the uncertainty around the real-world value and effectiveness of new approaches, for many, necessitates an iterative approach to monitoring a product’s benefit–risk profile throughout its life span. Global policy makers have been launching a number of coordinated strategies to drug development, licensing, and reimbursement, exemplified by UK’s Accelerated Access Review, the EU Adaptive Pathways pilot, and Japan’s Sakigake legislation.

Although there are no proven methods or established frameworks to reinvent a pathway for adoption of ATMPs, this level of system change will rest on new avenues, founded on continuous dialog and interactions between stakeholders not traditionally connected, from developers and regulators, to payers, patients, and funders. The growing space of global precompetitive consortia and public–private partnerships (PPPs) can illuminate some of the critical enablers needed for this level of engagement and coordination.

By deconstructing challenges that exceed the capacity of single organizations, national and global consortia linking government, academia, and industry, such as the EU Innovative Medicines Initiative (IMI) or the FDA Critical Path Institute (C-Path), have covered significant ground during a remarkably small window of time toward the development of new knowledge, translational tools, and infrastructure that advance the biomedical space. However, as we move toward higher complexity measures of progress, like the development and delivery of transformative therapies, these interactions have to transcend the scientific space, to devise new organizational and policy frameworks, build the infrastructure needed by health systems, and ultimately reduce the financial uncertainty in this space.

Against a backdrop of growing demands to drive meaningful patient outcomes from ATMPs, we have to become better in three critical areas: deploy the operational flexibilities offered by the variety of collaborations, upholding novel flexible policies and pathways to address the inherent gaps in quantifying long-term effectiveness, and building infrastructure and test-bed environments for knowledge development.

By reflecting on the successes and lessons learned from collaborations over the past two decades, this work aims to inform the foundations for a future roadmap that ensures ATMPs and important new treatments can reach patients, while delivering value to all stakeholders. This paper refers to various global examples of vehicles for cross-stakeholder dialog, as well as emerging accelerated development pathways, all of which will be paramount to maintain momentum and smooth the path to approval, reimbursement, and access, for ATMPs that are following on behind.

## Setting Sail: Entering the Era of ATMPs

After 30 suspenseful years, the field of ATMPs is finally coming of age, with clinical successes already emerging across diverse areas of unmet need, from oncology and cardiology, to vision repair and skin/tissue regeneration. In 2016 the first gene therapy in the EU was approved, GSK’s Strimvelis for Adenosine Deaminase Severe Combined Immunodeficiency (ADA-SCID), and is presently only reimbursed in Italy, whereas ChondroCelect, the first EU approved cell therapy, is still only covered in Spain, Belgium and the Netherlands. Although over 650 clinical trials have been conducted to date, only 8 ATMPs are granted a marketing authorization in the EU, with 2 withdrawn from commercial activities due to lack of uptake (3).

Upholding and replicating the successful stride of an effective treatment for a clinically challenging condition, like Adenosine Deaminase Severe Combined Immunodeficiency (ADA-SCID), would provide a much-needed technical, clinical, and commercial proof for the larger scale adoption of ATMPs, just like rituximab became the undercurrent for the advent of monoclonals. However, with the last three decades focused primarily on advancing our scientific understanding of ATMPs, a number of unique challenges remain as clinical knowledge, policies, skills, and services are still co-evolving with the technology in real time.

## Uncharted Waters: Canvassing Unique and Persisting Bottlenecks

Production of ATMPs involves the manipulation of living, cell-based materials (and viral vectors for gene therapies), all thus underpinned by distinctive variability. The sensitivity of these materials requires novel processes, complex development systems, and sophisticated quality control streams, calling for skills and infrastructure unlike anything used for traditional pharmaceuticals. Although vector gene therapies and products from standardized tissues are less time sensitive and can leverage more traditional supply models, for autologous products or where product shelf-life is limited, there is need for specialized centers for access and treatment, which can accommodate “bedside” closed systems and decentralized supply chains (4).

It is key to understand that in this space, the “process *is* the product”, as any change in manufacturing could affect a treatment’s efficacy and safety. This is a paradigm change in regulation, posing new riddles around Good Manufacturing Practice (GMP), requiring new standards for quality, potency and safety, as well as process design and assurance strategies (5). With individual batches essentially corresponding to a different product, ATMPs also face unique challenges in product standardization, including inspection and release testing (6).

These challenges are ever more important for production scaling-up from early phase 1 and 2 trials, currently done within small academic or hospital GMP facilities, to Phase 3 trials and commercial supply, to ensure product equivalence and cost control (7). Clinical development of ATMPs is also met with an inefficient assessment framework, failing to provide clear go/no-go decision criteria (8). ATMP trials are highly type and disease dependent, tailored to much smaller patient populations. Traditional algorithms are not adequate to capture the potential lifetime effects, calling for new endpoints and designs (i.e., for single-arm trials), which becomes more perplexing under the light of evolving knowledge around ATMPs (9).

With a number of scientific challenges yet to be resolved, the costs of ATMPs remain high. Moreover, the promise for lifelong effectiveness raises regulation and reimbursement challenges around the limited availability of evidence at the point of approval and pricing negotiations (6), as well as budget impact and affordability issues that shift influence from Health Technology Assessment (HTAs) to payers. Uncertainties around data availability and maturity question how ATMPs can meet cost-effectiveness thresholds in the existing HTA methodologies, which could disproportionally disadvantage them (10). Outstanding issues also include the discrepancy between evidence for regulatory approval and for HTAs, as well as harmonization of HTA requirements and methodologies across Europe and globally.

Despite the ongoing progress, ATMP development timelines are still long and winding and in addition to dealing with the regulatory complexity, developers, mostly SMEs, face huge risks in accessing capital, while meeting HTA requirements and negotiating coverage. Maintaining current momentum and investment in this nascent space will require funders to have increased clarity on a product’s journey to market and the views of regulators and payers (11). With established supply chains and assessment paths limited to traditional small molecules and large biologics, ATMP’s call for a reinvention of the entire pathway from production, to assessment and adoption (12).

## Changing Course: Reinventing the Way We Develop Treatments

Because of their promise for sustained effect and an individual–patient focus, ATMP discovery, development, manufacturing, and licensing/coverage assessment steps become less linear and predictive than traditional drug discovery and more co-located than established supply chains. Given the patient-targeting nature of the majority of ATMPs, manufacturing and quality aspects are also embedded from discovery through to development, while clinical assessment and adoption are seamlessly linked. Securing patients’ access to these therapies, thus, requires a more coordinated approach across product development and enhanced capacity for stakeholder collaboration (13).

Although ATMPs lend themselves naturally to a greater level of coordination, not all of these challenges are uncommon to other breakthrough areas (14). Despite the high investment in R&D during the past decades, or perhaps as a direct consequence, a striking gap remains in innovation reaching patients, as breakthrough science is outpacing the current assessment system in a number of ways (15). Growing patient demand for timely access to better treatments, new science leading to segregation of disease subtypes, and patient-tailored, precision medicines, as well as growing pressures for measures of budget impact and the value of new products, are common drivers of change that force new business incentives to keep innovation alive and sustainable. Simply securing regulatory approval for a new product is no longer an adequate marker of success. The yardsticks have moved, requiring novels ways to deliver new, better, affordable therapeutics to the right patients faster and do this reliably and sustainably.

The biggest challenge involves getting earlier/timely patient access, while equipping decision makers with adequate information on the benefit/risk thresholds. Without any prior clinical experience for ATMPs, where stability of the effect needs frequent reassessment, current systems focusing on upfront evidence to predict real-life drug performance, bear little relevance. Against this backdrop, acceptance of higher uncertainty can only be balanced by the real-time monitoring and continuous generation of development and treatment outcomes evidence, throughout the lifecycle of ATMPs. Arguably, the only sustainable access route to market and the patients involves re-engineering a transparent and coordinated approach to clinical assessment, licensing, and coverage, including monitoring of clinical use.

It is clear that the time has come to improve our innovation strategy. Progressing the ATMP space beyond early examples of clinical efficacy and toward adoption on a larger scale will require a set of important adaptations, predicated on early and continued efforts to remove barriers to collaboration. Practical solutions have to be developed within three key domains:

1. Maximizing use of emerging flexible tools on licensing and reimbursement.
2. Deploy the flexibilities offered by collaborations and develop new platforms for convergence.
3. Establish infrastructure and “test-bed” environments for capacity and knowledge building.

### Increasing Systemic Flexibilities for ATMP Adoption

Regulators were the first to step up to the challenge of balancing access under limited evidence by devising new ways to manage this uncertainty. In recent years, a number of flexible licensing pathways were introduced across the world to allow for accelerated access, provided that patient benefits outweigh the need for additional data (16). Notable examples include the MHRA Early Access to Medicines Scheme and the NHS Accelerated Access Review in the UK, the Adaptive Pathways pilot and the 2016 PRIME scheme in the EU, and the FDA Breakthrough Treatment Designation in the US (17) (Table 1).

**Table 1:** **Key examples of the existing and emerging pathways of relevance to Advanced Therapy Medicinal Products (ATMPs), covering regulatory, reimbursement, and access and new stakeholder dialog platforms in EU, US, and the UK as example of a national jurisdiction**.

	Existing tools	New and emerging schemes	Platforms to facilitate adoption
EU	• ATMP regulation • Emergency use, exceptional circumstances • Orphan designation • ATMP hospital exception • Scientific Advice, Protocol Assistance • Compassionate use for unlicensed drugs • Conditional Marketing Approval	• Accelerated assessment • Adaptive pathways pilot (lifecycle approach) • EU PRIME scheme on priority medicines **Reimbursement**: • Managed entry/patient access agreements	• European Medicines Agency (EMA) Innovation Taskforce: for academics and SMEs • STAMP from EC Expert Group • Parallel reviews: – EMA/HTA scientific advice – EMA/FDA review • Registries and other PHV tools; EMA Registries pilot

US	• Fast Track • Accelerated approval (with surrogates) • Priority review **Reimbursement** • Coverage with evidence development	• SMU; Special Medical Use for disease subsets • Breakthrough Therapy designation • Regenerative Medicine Advanced Therapy designation (RMAT) **Reimbursement** • Managed access for private payers	• FDA Critical Path Innovation Meeting • Parallel Scientific advice between EMA/FDA

UK	• Early Access to Medicines Scheme (EAMS) • UK Specials • NICE Scientific Advice mechanism	• Accelerated Access Review (AAR) • NHS Commissioning through evaluation • NHS Executive Specialized Commissioning schemes	MHRA Innovation Office Regenerative Medicine one stop shop^a^ Innovation Partnership: NHS, MHRA, NICE, NIHR NICE Office for Market Access NICE “mock” technology appraisal on CD-19 CAR-T

*The list is not exhaustive. ATMP regulation: (EC) No. 1394/2007*.

*^a^The UK’s One Stop Shop: includes MHRA, the Human Tissue Authority, The Human Fertilisation and Embryology Authority (HFEA), and Health Research Authority (HRA). For a detailed analysis or comparison of these schemes, please see Ref. (16, 18–20)*.

In the same spirit, the reimbursement space saw the advent of more iterative approaches that allow the gradual buildup of evidence, including managed entry agreements and coverage with evidence development (21). These also mark a shift from an one-off view on payment assessments to progressive schemes to measure product value and reduce uncertainty around cost-effectiveness at the time of negotiation (18). ATMP affordability discussions have also led to proposals for risk-sharing schemes (i.e., lifetime leasing or annuity-based models) that would allow a more adaptive way to gain evidence on anticipated value (19).

For ATMPs, whereby long-term effectiveness is difficult to quantify at the outset, schemes that balance acceptance of uncertainty with a preagreed and clear plan for progressive knowledge accumulation can be truly transformative. Built on the premise of early and continued stakeholder cooperation, in 2014 the European Medicines Agency (EMA) launched a pilot on the Adaptive Pathways scheme, setting the foundations for novel coordinated pathways from clinical assessment to HTA (20). The scheme poses an iterative development program that allows early approval and coverage for a benefit/risk optimized population through ongoing evidence gathering, often exploring the use of smaller trials and surrogate endpoints (22).

By allowing earlier clinical use, such adaptive approaches for development would press forward the confirmation of a product’s real-world performance and provide much-needed clarity on downstream coverage criteria, urgently sought by ATMP investors and manufacturers. They also provide a key opportunity to align and address the evidence requirements for licensure and reimbursement, subject to stakeholder connectivity around post-authorization commitments and the continued collaboration between manufacturers, regulators, HTAs, and payers, as well as patients.

### Advancing Collaboration for Advanced Therapies

#### The Common Language of Innovation: Deploying the Tools of Open Innovation

The challenges in reinventing an adoption route for ATMPs are as much about the scientific and clinical components, as they are about the organizational structures. Early and sustained interactions between academics and manufacturers, regulators, HTA assessors, and patients will be critical to start aligning, at least some, aspects of the decision-making process and leverage the progressive accumulation of new knowledge on benefit/risk that emerging translational tools and digital infrastructure allow. As pressure to deliver transformative treatments increases, many seek to understand how to establish the environments necessary for stakeholders to share resources and risk and achieve goals as complex as the emergence of a sustainable ATMP sector.

Over the last 20 years, collaboration models, such as “public–private partnerships” (PPPs) and “precompetitive consortia,” have grown in popularity in the global pharmaceutical industry in response to complex biomedical challenges (23). This broad definition covers a diverse range of structures across disciplinary, organizational, stakeholder, and geographic boundaries, from PPPs like the US C-Path, the Foundation for NIH (FNIH), and the EU IMI, to open-source collaborations like the Structural Genomics Consortium and Sage Bionetworks (24), or industry safe havens like TransCelerate (25) (Table 2). In the field of ATMPs, the various banking initiatives on stem cells for preclinical, clinical, and pharmacological work are central stage, including the EBiSC and StemBANCC IMI projects, as well as the Human-Induced Pluripotent Stem Cells Initiative (HipSCi) in the UK.

**Table 2:** **Drug development stage classification of biomedical collaborations: examples of consortia addressing different stages of the value chain and further information [adapted from Papadaki and Hirsch (26)]**.

Innovation level	Collaborative goal	Example	Deliverables
New translational enablers, novel technologies	Increase R&D predictive capacity	Biomarkers Consortium: www.biomarkersconsortium.org/?	Biomarker identification and qualification

	Open-source molecular data gathering and analysis on human disease	Sage Bionetworks: http://sagebase.org/	Technology networking infrastructure; governance policies; disease models

	Increase R&D predictive capacity; safety	International Serious Adverse Event Consortium (iSAEC): www.saeconsortium.org/?	Identify biomarkers that predict the risk of drug-related serious adverse events

	Accelerate development of new drugs	Coalition Against Major Diseases (CAMD): www.c-path.org/camd.cfm	Technologies and tools in drug development for neurodegenerative diseases

Development process optimization	Improve clinical trial efficiency	iSPY 2: http://www.ispytrials.org/home	Advance regulatory standards for novel clinical trials designs and personalized medicine

	Ensure quality of biomanufacturing processes	Biomanufacturing Research Program (BIOman): http://cbi.mit.edu/research-overview/bioman/?	Manufacturing and quality control of biopharmaceuticals

	Improve clinical trial quality and efficiency	Clinical Data Interchange Standards Consortium (CDISC): http://www.cdisc.org	Data Standards and healthcare information

Approval and market access	Advance adaptive development, patient access, post-market learning paradigm	NEWDIGS: http://cbi.mit.edu/research-overview/newdigs/?	Simulation methods/tools; facilitate pilots and knowledge sharing across global jurisdictions

	Advance methods, policies for observational/outcomes research to enable coverage with evidence development	Center for Medical Technology Policy (CMTP): www.cmtpnet.org/?	Clinical research standards, infrastructure, and coverage/reimbursement policy

New business models	Fund late-stage health technologies for the developing world, maximizing returns in mature markets	Global Health Investment Fund	Risk protection for investors; venture funding for late-stage technologies

Disease specific	Cure Parkinson’s	Michael J. Fox Foundation	

	Oncology	Cancer Commons: www.cancercommons.org/?	Targeted treatments for patients with cancer

All of the above	Provide ongoing infrastructure and funding for collaborative EU-wide innovation	Innovative Medicines Initiative (IMI) 1 and 2: http://www.imi.europa.eu/	80+ consortia

	Increase drug product development efficiencies by identifying pathways to integrate new scientific advances into the regulatory process	Critical Path Institute: https://c-path.org/programs/	14 consortia

The efficient deployment of partnerships has become a key competency of the healthcare system, leveraging their flexibility to deconstruct complex challenges into manageable work streams to achieve shared outputs (27). Their significant progress in producing a range of enabling tools, platforms and new processes that advance drug discovery and development, has been extensively documented (28). Moreover, the experience of major consortia on novel governance, IP policies, and other operational models provide valuable lessons for new initiatives (29). Perhaps most importantly, precompetitive consortia have generated safe havens for transparent sharing and alignment, allowing different stakeholders to build intellectual and working proximity and interact in ways not previously possible (30).

With more than 400 consortia estimated to operate globally (31), growth in the number of narrowly scoped collaborations has led to challenges in their coordination, oftentimes seen as duplication, fragmentation, and consortium fatigue (26). In addition, while many have successfully delivered their target outputs, defining their impact on the delivery of better treatments remains elusive, requiring the combination of outputs from different collaborations, each working on some aspect of the development and access pathway. Looking across the diverse range of consortia successes, examples such as standards development and the validation of new tools typify the next level of challenges that go beyond scientific collaboration, having to address additional regulatory processes and barriers.

#### From Collaboration to Transformation: Redefining Value

The biomarkers and clinical endpoints resulting from collaborations like the Biomarkers Consortium (BC),^1^ AD Neuroimaging Initiative,^2^ the C-Path Predictive Safety and Toxicology Consortium,^3^ and Coalition Against Major Diseases (CAMD), as well as numerous IMI consortia, are key examples whereby profound regulatory qualification gaps have been limiting their utility (32). In the clinical trial space, however, the BC has laid out a path for the incremental deployment of its biomarker and knowledge outputs, to inform policy changes that subsequently advance new genomic-driven clinical trial designs. Expanding the work of the I-SPY1 consortium on clinical endpoint validation, the follow-on I-SPY2 trial in oncology used “master IND” approach to support multi-asset submission and co-development of diagnostics. With I-SPY2, the BC pushed beyond the adoption of single product-focused biomarkers to inform entirely new adaptive trial practices and regulations that can apply across assets and diseases, revolutionizing current investigational approaches in oncology (33).

**Figure 1:**
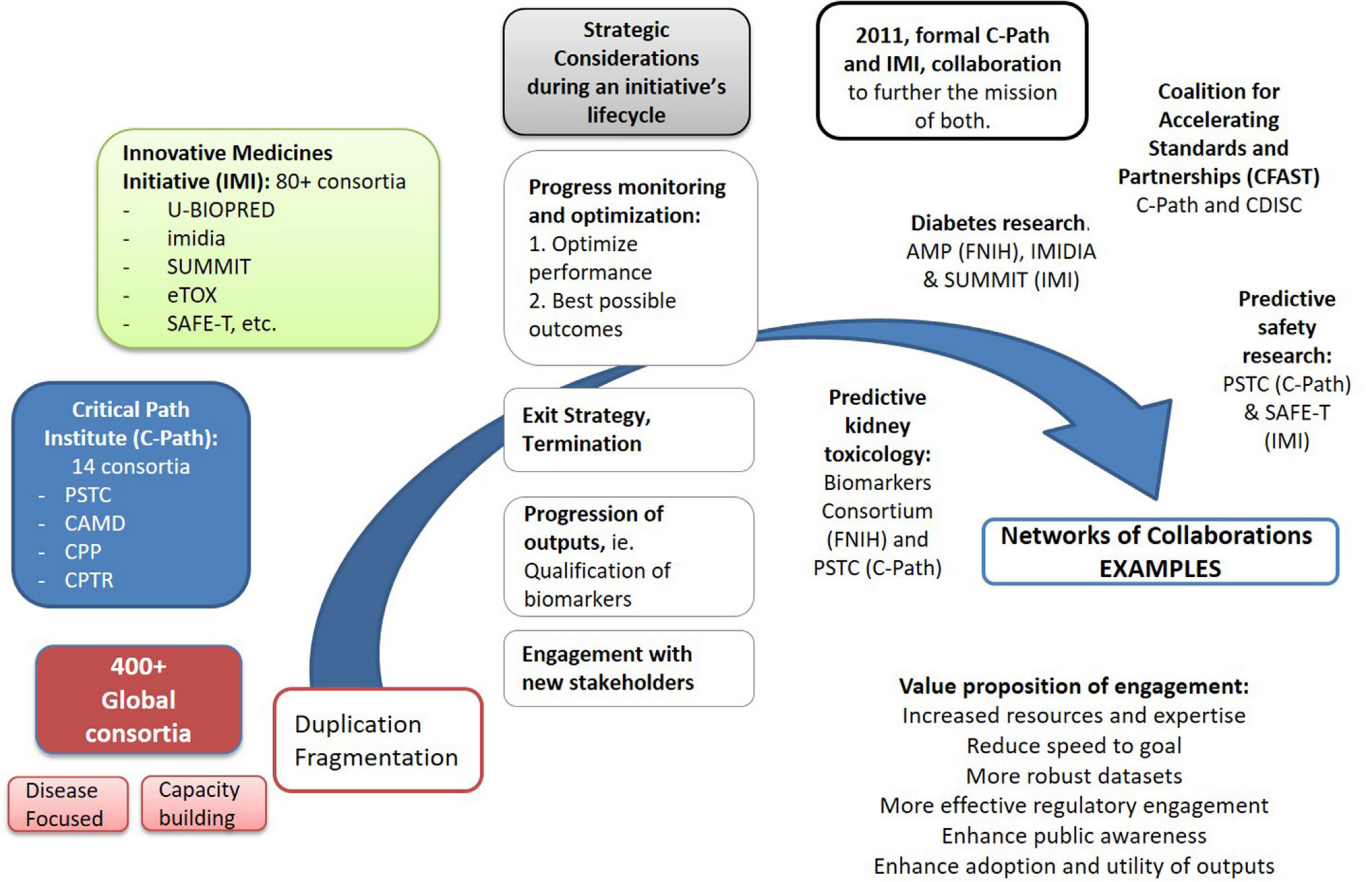
**Driving collective impact requires an increasing coordination of activities across global consortia, as well as individual organizations, to reduce duplication of efforts and maximize impact from the use and adoption of their initial separate outputs**. A growing number of strategic interactions among the global ~400 partnerships are already emerging. Spearheaded by the formal collaboration between the FDA Critical Path Institute and Innovative Medicines Initiative (IMI), signed in 2011, a number of linkages have been formed among several of their distinct consortia in diseases like Alzheimer’s [Pharma-Cog^1^ and EMIF^2^ working with Coalition Against Major Diseases (CAMD)], tuberculosis (PreDiCT-TB^3^ and CPTR^4^), or broader fields like Predictive Safety and Toxicology Consortium (PSTC) (C-Path) and SAFE-T (IMI) in preclinical safety research. A number of global consortia have also joined forces with other initiatives pursuing relevant activities to avoid duplication of their efforts. Notable examples include the partnership of the Accelerating Medicines Partnership (FNIH) with IMIDIA/SUMMIT (IMI) in diabetes, the Biomarkers Consortium (FNIH) with PSTC (C-Path) on the kidney safety project, or in broad fields like data standards, with C-Path and Clinical Data Interchange Standards Consortium (CDISC)^5^ forming Coalition For Accelerating Standards and Therapies (CFAST),^6^ or CDISC and CAMD working in partnership. As the complexity of biomedical challenges increases, it will be important for initiatives to envision early in their lifecycle the strategic connections that may be needed to explore new combinations of their deliverables and resources and engage additional decision makers, ultimately decreasing uncertainly across the path from basic discovery to patient care. ^1^http://www.imi.europa.eu/content/pharma-cog. ^2^European Medical Information Framework, http://www.imi.europa.eu/content/emif. ^3^http://www.predict-tb.eu/. ^4^Critical Path to TB Drug Regimens: http://c-path.org/programs/cptr/. ^5^Clinical Data Interchange Standards Consortium (CDISC); http://www.cdisc.org. ^6^C-Path Coalition for Accelerating Standards and Therapies; http://c-path.org/programs/cfast/.

In response to the above challenges, several collaborations set out to form strategic connections to drive incremental value from their separate outputs, shown in Figure 1. This transition highlights the importance of reaching for more strategic measures of progress for collaborations on the broader context of enhancing the adoption of innovation, through strategic connections between different consortia to explore new combinations of their delivarables and resources and engage additional decision makers.

#### The Evolution of Engagement Models

For ATMPs, collaboration will undoubtedly be a game changer. The goals of partnerships, however, should exceed beyond good science, to target innovation across the pathway to patients and the interdependent domains of regulation, policy, and human capital development. The stakes are up, calling for increased capacity to use the full spectrum of open innovation and collaboration platforms currently emerging to generate value streams that exceed the traditional, linear model of pharmaceutical development.

Besides the “bricks-and-mortar” collaboration structures typified by the global PPPs and precompetitive consortia, a number of less structured platforms for dialog and interactions also emerged in recent years, integrating and coordinating the activities of different stakeholders and providing additional organizational models to capture, assess, and apply emerging knowledge and outputs in the translational system. Earlier dialog between regulators and payers, as well as developers, has been enabled by the number of Innovation Offices launched recently, from the MHRA Innovation Office and the NICE Office for Market Access in the UK, to the EMA’s Innovation Taskforce and the FDA Critical Path Innovation Meeting in the US (Table 1). In the regulatory space, by working early with all key global health authorities, the NEWDIGS consortium of MIT was able to generate a series of scenario design exercises on real assets that informed the EMA’s eventual pilot launch of the Adaptive Pathways pilots (34).

Similarly, groundwork for more coordinated dialog is already laid out between EMA and EUnetHTA in Europe, and the parallel assessment pilot between FDA and Medicare in the US (35). Moreover, the EU has been deploying a number of initiatives to limit the gap between market authorization and technology assessment, such as AdHopHTA, Advance_HTA, and INTEGRATE HTA that further exemplify the value of safe haven environments in the exploration and development of alignment on key trade-offs for decision-making and follow-on policy.

#### More Than the Sum of Parts: New Coordination Activities

Taking stock of the progress of global initiatives can illuminate how the follow-on connections between different partnerships or stakeholder groups starting to shape can accelerate both product and process optimization. The complex challenges of ATMPs will require many of these initiatives working on some aspect of the value chain to come together through strategic connections to explore new combinations of their outputs. An important step in this direction will also involve making full use of the flexible discussion environments and taskforces currently emerging globally, which aim to bring together all key players across the value chain, not least regulators, payers, and the patients.

The US is setting an early example in cancer treatments. The National Immunotherapy Coalition brings together large pharma and biotech companies, major academic cancer centers, payers, and financial institutions to turn combination immunotherapies into the next standard of care in cancer (36). The US National Cancer Institute has also joined forces with oncologists and academic medical centers to launch an impressive number of trials to test drug combinations tailored to individuals’ immune profiles (37).

Europe’s IMI 2, the largest global PPP in the life sciences, has a unique armamentarium of projects targeting different aspects of science and development. The next chapter for ATMPs presents it with an opportunity, and challenge, to identify convergence points between its various initiatives and develop a new model environment for coordination. The EBiSC and STEMBANCC initiatives working on stem cell banks, reference materials, and new standards have significantly increased confidence in the feasibility of cell therapies and could further derisk emerging areas, such as the application of genome editing (38). Planning for the linkage of such consortia could advance the value of their deliverables, allowing for the development of common standards on banking criteria, cell-type definitions, or the harmonization of cultivation protocols toward the comparability of data and cells between different banks or activities outside of IMI2, like HipSCi in the UK (39). A number of the broader IMI consortia have also been focusing on the underpinning infrastructure for emerging paradigm reforms, like the use of real-world data (GetREAL), the development of patient and disease registries (i.e., The CSA for Big data for Better Outcomes), or the Enabling Platform on Medicines Adaptive Pathways to Patients (ADAPT-SMART).

**Figure 2:**
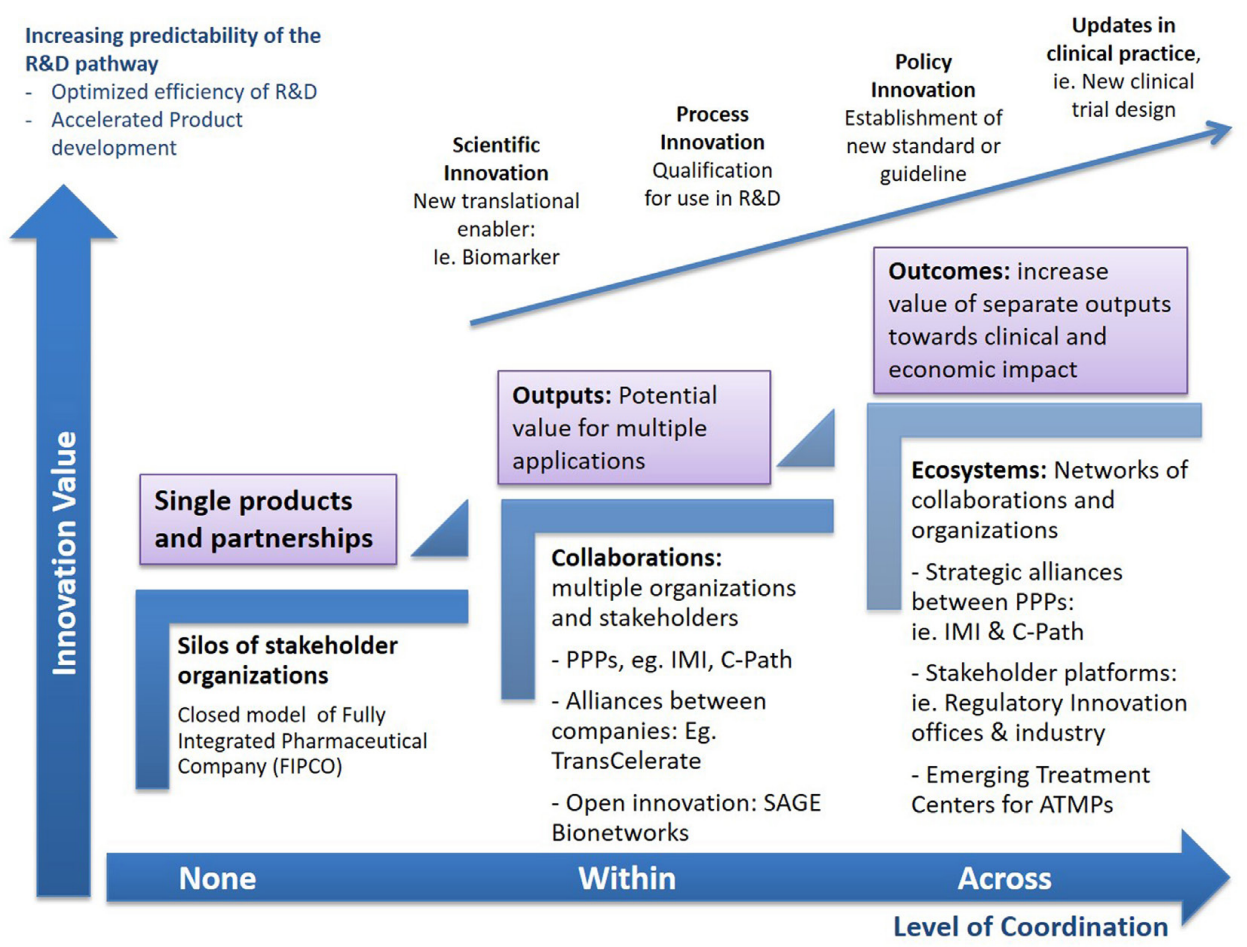
**Increasing impact from collaborations**. The complex challenges of Advanced Therapy Medicinal Products (ATMPs) will require many initiatives working on some aspect of the value chain to come together through strategic connections to explore new combinations of their outputs, converge emerging knowledge, or launch pilots on new challenges within established projects. Through strategic follow-on connections between different consortia, as well as collaborative platforms that allow the convergence of multiple stakeholders, the value of individual deliverables and outputs can be augmented and expand beyond the initial projects, disease areas, or R&D stages, aiming to advance the entire pathway to patients. Multiple routes to synergy may exist for any single consortium, from piloting the use of a new enabling tool, i.e., biomarker, in a research protocol or clinical trial, to customizing data standards for regulatory application or ultimately propelling formal policy and process updates. Accelerating the development and delivery of advanced therapies requires collaborations to move toward higher complexity measures of progress that transcend the scientific space, to devise new organizational and policy frameworks, accelerate product delivery, and maximize process efficiencies.

Evidently, the transition from specific outputs to patient-level outcomes is not straightforward (40). One-size-fits-all solutions are unlikely and a range of connections between infrastructures at the global/local and private/public space are wanted to extract add-on value from collaborations, capture knowledge across the differed stages of R&D, and use these to inform the practice and policy updates ultimately decreasing uncertainly across the path from discovery to patient care (Figure 2).

### Developing Enabling Infrastructure and Test-Beds

Although both the emerging development paradigms and the successful stride of global consortia are important signs of progress toward getting better drugs to patients, challenges for ATMPs are ever more perplexing. The level of mass customization of production, administration specialization, and coordination of development stages that these therapies demand is unmatched. Conceivably, the specialized and bespoke solutions needed likely reside outside traditional company boundaries, further pointing out that strategic partnerships and open innovation could play a significant role in exploring these novel possibilities.

Without prior clinical experience, the shift from traditional predictive approaches to real-time monitoring of development and treatment outcomes is necessary to increase the robustness of benefit/risk knowledge and inform subsequent requirements. The use of single-arm trials will require data from historical or disease-specific control populations (41), whereas HTA evaluations through patient outcomes would also require the inclusion of cost-effectiveness criteria in ATMP clinical trials (42). Long-term follow-up of patients is paramount in controlling safety and efficacy concerns and similarly, the high degree of batch specificity of single products, also calls for new quality and tracking systems and data infrastructure.

Evidently, technical and historical data and real-world evidence (i.e., from registries, hospital exception, and compassionate use records) become the connective thread between research, development, patient access, and commercialization. For an unobstructed access to ATMPs, data utilization and the design of suitable collection frameworks to monitor safety, effectiveness, and epidemiological endpoints become indispensable in providing stakeholders with sufficient decision-supporting evidence on the use of these therapeutics in real-life conditions. Yet, compensating for the uncertainties of non-conventional ATMP development requires the combination of novel information sources and data-aided technologies that span beyond the capacity of single organizations or stakeholder groups.

A matched effort for innovation from the bench to the patients is indispensable in ATMP manufacturing, implicating all actors across the supply and value chain. Starting from patient material sourcing and control to the strict GMP requirements, the role of specialized facilities from collection to final product administration is obligatory. For gene therapies, manufacturability is a consideration as early as vector design (Quality by Design, QbD), requiring the advent of novel fast, accurate, and robust analytics, whereas ongoing progress in process development and quality assurance presses regulators to step up the quest for new, written, and practice standards.

#### ATMP Specialized Centers: New Platforms for Data-Enabled Decision and Risk Sharing

However, marrying the high cost of developing and manufacturing these treatments with the growing trends for affordability, further exacerbated by low patient indications, can prove a showstopper for many, requiring that we deliver change in two critical aspects. The first involves the earlier utilization of advanced therapies in real-world patient settings. The second, the implementation of networked activities between multiple manufacturing and clinical delivery platforms and processes, enabled by greater proximity and collaboration between the different players across the development and supply chain.

On the former, meeting the requirements for earlier patient and market access might be easily achieved through appropriate control of access and prescribing, suggesting that treatment areas needing specialized centers for diagnosis, treatment, and patients’ follow-up would be good places to start. This is not far from the current reality for ATMPs, with primary targets still limited to rare or highly genetically defined indications, such as immunodeficiencies, hematologic, and metabolic diseases.

For ATMPs, specialized treatment centers also have to combine administration to patients with capabilities for clinical testing and commercial manufacture in an in-hospital setting, requiring manufacturing units and specialized contractors, academic research and clinical centers, as well as the patient bedside to become development partners with health systems. These networked clinical environments can also spearhead novel business models, where decision-making, cost and risk of establishing efficacy, safety, and quality are being shared and enabled though an infrastructure that links data across stakeholders and stages of development. Systems for continuous patient monitoring can in turn increase our understanding of the molecular underpinnings of disease and treatment response, for example, by enabling the profiling of vector integration patterns across the preclinical and clinical studies of a gene therapy.

Enabled through a shared data infrastructure and aligned decision-making, establishment of such hospital-centered development and access models will kick-start the real-world use of these treatments, allowing an earlier collection of evidence on clinical and cost-effectiveness, and most importantly secure patient delivery. It will also build capacity for clinical manufacturing and formulation of ATMPs at scale and help the development of the accompanying supply chains and logistic support.

**Figure 3:**
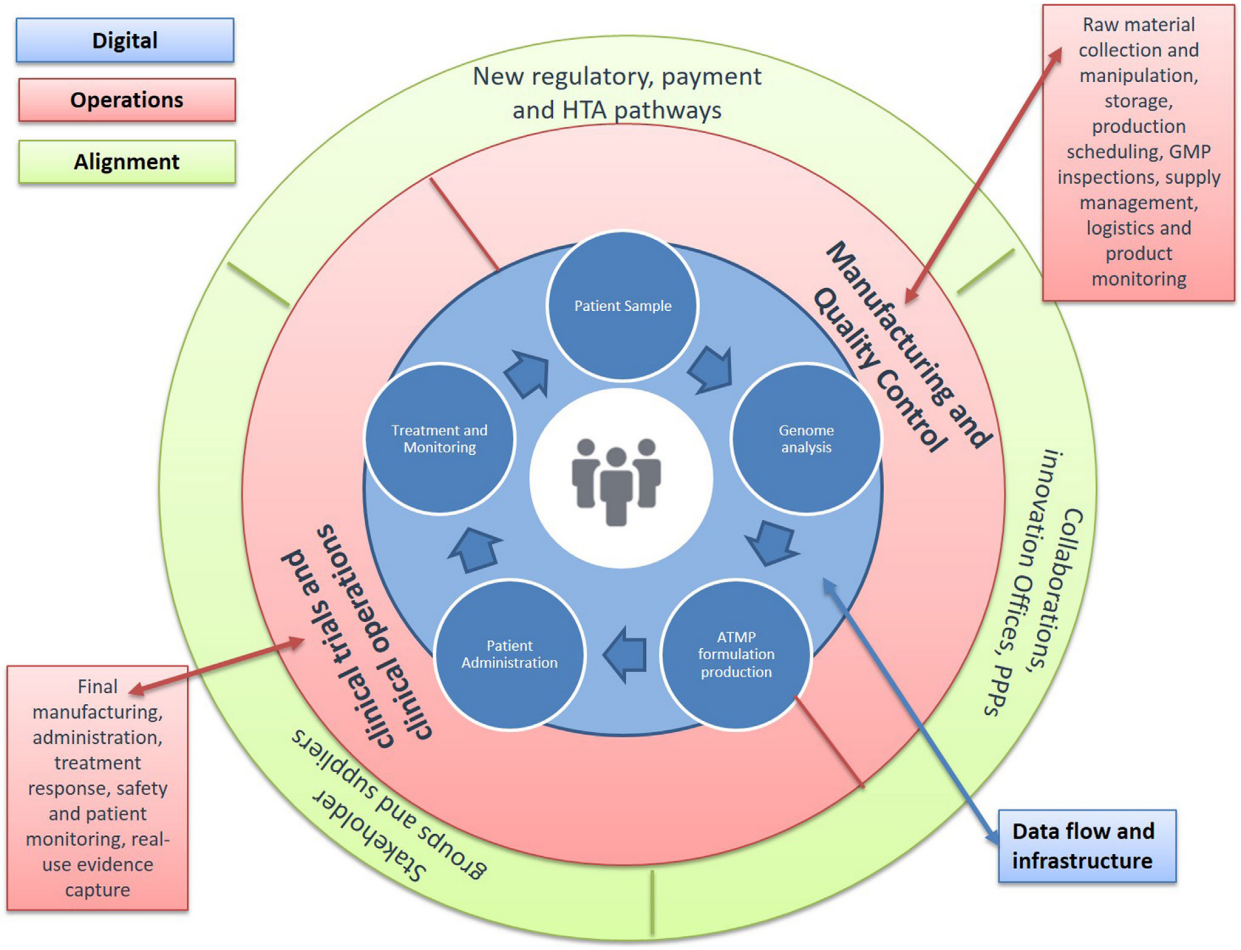
**Advanced Therapy Medicinal Product (ATMP) Treatment Centers**. Developing new environments for decision, cost, and risk sharing. A number of barriers in the space continue to keep production costs high and further challenge licensing and reimbursement success possibilities. Solutions are urgently needed across the continuum from discovery to patient access: (1) new approaches to monitor and improve manufacturing quality, including transfer of production from academia to Good Manufacturing Practice (GMP); (2) availability of standardized tools to aid regulatory decisions around efficacy, safety, and quality; (3) novel clinical trial designs that allow for small patient numbers and outstanding variability; (4) methodologies to understand cost-effectiveness and access; and (5) data structures to improve product use and health outcomes. Achieving these requires changes in two critical aspects: allowing the earlier clinical use of ATMPs and building the necessary environments for the progressive generation of data across the path of new products to the clinic. A key step in this direction would be the establishment of specialized treatment centers for ATMPs that enable networked activities between manufacturing units and specialized contractors, academic research, and clinical centers, as well as the patient bedside. These networked clinical environments would combine administration to patients with capabilities for clinical testing and commercial manufacture in an in-hospital setting. They would also allow for new business models, where decision-making, cost and risk of establishing efficacy, safety, and quality are being shared and enabled though an infrastructure that links data across stakeholders and stages of development. They also provide a key opportunity to address the evidence requirements for licensure and reimbursement, allowing greater stakeholder connectivity around post-authorization commitments.

So far, ATMP manufacturing has been largely residing within academia and the research space, bearing little GMP congruence and limiting capacity for clinical and commercial transferability. Enabling interactions between the developers of new products with the clinical facilities, as well as manufacturers of novel tools and platforms, can propel the industrialization of technology innovations and their adoption in the clinical setting (Figure 3). This can be a significant gain, given that the current costs of ATMP production systems are still affecting their early adoption, which could lead to significant regulatory hurdles and comparability validation work, if they are used later in development. The potential of these clinical centers to accelerate the development of technologies that reduce the cost and increase the efficiency of ATMPs also represents a tremendous opportunity, providing a route for smaller companies and Contract Manufacturing Organizations to engage with the space to develop, prototype, and qualify the equipment currently missing.

The frontman of gene therapy, GSK’s Strimvelis, was developed in partnership with Italy’s San Raffaele-Telethon Institute for Gene Therapy (TIGET) in Milan, which is also the therapy’s only point of access. A joint venture between the San Raffaele Hospital (SR) and the Telethon Foundation, TIGET, comprises an early example for these ATMP specialized centers. Its networked model enables the coordinated design of new therapy approaches, combining Telethon’s specialization around gene transfer, genetic modification, and preclinical models of stem cells, with the translational and clinical units, as well as expertise of SR-hospital in regulatory and clinical testing.

From a country’s perspective, TIGET’s access exclusivity on Strimvelis (43) also shows that these centers could turn into global hubs for ATMP commercialization, further strengthening local health economies, attracting investment and propelling job creation and economic growth. Countries like the UK, with strong-networked foundations already in place, could also capitalize on the current momentum. With a number of excellent GMP facilities across its academic institutions, hospitals, the NHS Blood and Transplant service, as well as commercial players, ongoing national digital initiatives (NHS, Genomics England), and a unique network of Catapult Centers of excellence, the UK could set a global example on industrializing research and development and promoting sector growth for ATMPs.

The UK can give a valuable example of the strategic approach needed in the space, being at a strong position with substantial scientific progress, growing investment appetitive and reforms in its regulatory, reimbursement, and health system currently underway. If it is to deliver its promise to become global leader in the development and delivery of ATMPs, it must acknowledge the challenge of collaboration among all relevant actors, providing funding to support both basic and applied research and developing a sustainable and viable pathway for these products from bench to the bedside. Within its newly launched AAR on accelerated development routes for transformative products, in particular, the early consideration of ATMP challenges could prove instrumental in identifying critical factors for novel systems for assessment, commissioning, and patient access.

## Pirates in the Navy: The Essentials of ATMP Business Innovation

With ATMPs, the bet now is to turn innovations that once where unimagined, into treatments that we will not live without. In a space that combines an unprecedented level of technological progress with the need for systemic reinvention, successful companies will have to turn themselves into innovation powerhouses, radically changing the way they look at structures, teams, and people. As one of the world’s biggest innovators, Steve Jobs, once said “it was more fun to be a pirate than to join the navy.” ATMPs mark a revolutionary shift from closed biomedical strategies toward collaborative and networked innovation, enabled by the establishment of treatment centers, new supply chain, and business relations. From a company’s perspective, understanding how to operate in these new environments will be imperative, requiring an increased ability to deploy collaborations and their networks more efficiently.

As decision-making across the path to the clinic becomes more constant, ATMP developers must also build new “fit-for-purpose” business models that can leverage the adoption reforms underway globally and allow them to plug into the evolving landscape of stakeholder partnerships and networks. An overhaul in organizational practices is in order, if biopharma companies are to meet this next wave of therapeutics innovation, requiring the establishment of bespoke cross-functional teams from the R&D, clinical and regulatory, HTA/pricing and reimbursement, benefit/risk assessment, as well as business development and legal functions. Even with new organizational blueprints, the most important success factor remains the human capital. The next wave of connected innovation, branded by ATMPs, will require access to a new generation of healthcare leaders with capabilities to design new end-to-end pathways, skills at the interface of cutting-edge technology and commercialization, and ability to work across division and project boundaries.

## Land Ahead? Planning a Future Proofed Strategy for ATMPs

So, how are we doing in terms of building and growing this potentially transformative new treatment area? Taken together, the evolution in scientific understanding, new policy frameworks, and an increasingly collaborative health environment are key signs of progress in our ability to manage uncertainties in ATMP development. However, when it comes to having confidence in the capacity of current health systems to adopt these new treatment paradigms for the benefit of patients, the jury is still out.

Attaining complex measures of progress, such as the delivery of the wave of increasingly elaborate products like ATMPs to patients, involves moving away from the current binary go/no-go model of assessment to a life span approach to monitoring a product’s benefit–risk profile. This fundamental shift in the way we develop and test these products, rests on an unparalleled level of openness to early and continuous interactions between unfamiliar bedfellows, from industry and regulators, to payers and patients.

The recent growth in the global landscape of precompetitive collaborations and open innovation consortia introduced a new level of organizational flexibility, allowing the combination of stakeholder resources, knowledge, and objectives. The complex challenges of ATMPs will require many of these initiatives working on some aspect of the development pathway to explore new combinations of their outputs through strategic connections and allow the exploration of disease mechanisms, integrate dispersed knowledge around therapeutic approaches, and address crosscutting technical and clinical issues across development stages and treatment areas.

Taking stock of the progress of global initiatives can illuminate how follow-on connections between different partnerships or stakeholder groups can inform the establishment of a strategy that balances this flexibility with greater coordination within this diverse nexus of players and their networks. The development of new management and organizational infrastructure will be pivotal in driving and coordinating collective efforts within and across collaborations, and ultimately bring closer all stakeholders, not least regulators, payers, and the patients.

With the advent of ATMPs, the era of ecosystem-level innovation is on our doorstep, accenting these rapid changes and requiring that we continue to develop our collective capacity in two critical and synergistic directions. Removing perceived barriers to collaboration through new test-bed environments and connection platforms, and delivering a strategic roadmap that joins up the pathway from basic discovery to the market and the cycle of care.

Against this backdrop, the development of a knowledge base on the organizational frameworks needed to drive the evolution of collaborative innovation will also be important. Complementing our growing understanding of human health and disease with key principles from sociotechnical fields, including open (44) and distributed innovation (45), network theories (46, 47), systems thinking, and complex adaptive systems (48), among others, can provide useful insights on how to build up value from the growing global landscape of collaborations.

Delivering the promise of advanced therapies to tackle, and even cure disease, depends on our collective ability to effect an unprecedented level of change, through initiatives that target scientific challenges, alongside solutions in policy, regulation, business, and funding strategies. Demanding as this may prove, the pressure is on for everyone within our global healthcare systems, and especially those with life threatening or debilitating, unmet needs.

## Author Contributions

I am the only author of this work, executing all research, manuscript, and figure preparation.

## Disclaimer

The views and opinions expressed in this article are those of the author and do not necessarily reflect the official policy or position of any current or former affiliations.

## Conflict of Interest Statement

The author declares that the research was conducted in the absence of any commercial or financial relationships that could be construed as a potential conflict of interest.

## Footnotes

^1^http://www.biomarkersconsortium.org/.

^2^Alzheimer Disease Neuroimaging Initiative (ADNI): http://www.adni-info.org/.

^3^C-Path Predictive Safety Testing Consortium (PSTC): http://c-path.org/programs/pstc/.

## Abbreviations

ATMP, Advanced Therapy Medicinal Products; IMI, Innovative Medicines Initiative; C-Path, Critical Path Institute; PPPs, public–private partnerships; CAMD, Coalition Against Major Diseases; CDISC, Clinical Data Interchange Standards consortium; CFAST, Coalition For Accelerating Standards and Therapies; ADNI, AD Neuroimaging Initiative; BC, Biomarkers Consortium; FNIH, Foundation for the National Institutes of Health; AMP, Accelerating Medicines Partnership; CMOs, Contract Manufacturing Organizations; ADA-SCID, Adenosine Deaminase Severe Combined Immunodeficiency; PSTC, Predictive Safety and Toxicology Consortium; HipSCi, Human-Induced Pluripotent Stem Cells Initiative; EMA, European Medicines Agency; HTA, Health Technology Assessment.
